# Clinical Efficacy of Platelet-Rich Plasma and Hyaluronic Acid Versus Hyaluronic Acid for Knee Osteoarthritis with MRI Analysis: A Randomized Controlled Trial

**DOI:** 10.3390/jcm14103553

**Published:** 2025-05-19

**Authors:** Mandy Zhang, Kelvin Chew, Patrick Goh, Mon Hnin Tun, Kenneth Sheah, Victor Tan, Baoying Lim, Chung Sien Ng, Benedict Tan

**Affiliations:** 1 SingHealth Duke-NUS Sport and Exercise Medicine Centre, Changi General Hospital, Singapore 529889, Singapore; kelvin.chew.t.l@singhealth.com.sg (K.C.); victor.tan.a.k@singhealth.com.sg (V.T.); chung_sien_ng@cgh.com.sg (C.S.N.); benedict.tan.c.l@singhealth.com.sg (B.T.); 2 Sports Medicine International, Singapore 258500, Singapore; patrick.goh@ortho-intl.com; 3 Health Services Research, Changi General Hospital, Singapore 529889, Singapore; mon.hnin.tun@singhealth.com.sg; 4 Imaging Consulting, Singapore 217562, Singapore; ksheah@gmail.com; 5 SportsIN Orthopaedic Clinic, Singapore 258499, Singapore; limbaoying@gmail.com

**Keywords:** biological products, injections, intra-articular, cartilage, articular, bone marrow diseases

## Abstract

**Background:** Some evidence suggests that combining hyaluronic acid (HA) with platelet-rich-plasma (PRP) may offer synergistic benefits by enhancing the biological and mechanical properties of joints. However, data on the combination of HA+PRP vs. HA alone in the management of knee osteoarthritis (OA) remain limited. **Methods:** A double-blinded randomized controlled trial was conducted at an outpatient clinic and enrolled 58 patients with Kellgren–Lawrence grade 2–3 knee OA. They were randomly allocated to receive either intra-articular PRP combined with HA (*n* = 29 knees) or HA alone (*n* = 29 knees). The primary outcome was pain, assessed using a visual analog scale (VAS). Secondary outcomes included the Western Ontario and McMaster Universities Osteoarthritis Index (WOMAC), health-related quality of life (EQ-5D-5L), and structural changes on MRI, measured by the Whole-Organ MRI Score (WORMS). The VAS, WOMAC, and EQ-5D-5L were evaluated at baseline and at months 1, 3, 6, and 12. MRI WORMS was assessed at baseline and 12 months. **Results**: The baseline characteristics were comparable between the HA+PRP and HA groups. Both interventions showed improvements in pain and function at 12 months. However, the between-group difference in VAS at 12 months—the primary outcome—was not statistically significant (*p* = 0.102) and did not exceed the minimal clinically important difference (MCID) of 20 mm. The HA group demonstrated significantly greater VAS score reductions at 1 month (−31.1 [95% CI: −38.9 to −23.2] vs. −14.3 [95% CI: −22.2 to −6.4], *p* = 0.003) and at 6 months (−32.1 [95% CI: −40.1 to −24.1] vs. −19.2 [95% CI: −27.1 to −11.3], *p* = 0.024), compared to the HA+PRP group, although these differences did not reach clinical significance. No significant between-group differences were observed in the WOMAC scores, EQ-5D-5L, or total WORMS scores at all time points (*p* > 0.05). At 12 months, MRI assessment revealed a significant decrease in bone marrow edema in the HA+PRP group (−0.7 [95% CI: −1.6 to 0.2]) compared to the HA group (0.7 [95% CI: −0.2 to 1.6], *p* = 0.030). **Conclusions**: Both HA+PRP and HA treatments were effective in reducing pain and improving function in patients with knee OA over 12 months. While HA demonstrated greater early pain relief, the addition of PRP was associated with a significant reduction in bone marrow edema at 12 months. These findings suggest potential structural benefits of HA+PRP, although clinical superiority over HA alone was not established.

## 1. Introduction

Knee osteoarthritis (OA) is a prevalent degenerative joint disease that affects the tibiofemoral and patellofemoral joints, subchondral bone, and adjacent soft tissues [1]. This condition is characterized by musculoskeletal pain and functional limitations, which can significantly impair daily activities and impact quality of life [2]. Risk factors for the development and progression of knee OA include age, obesity, joint malalignment, previous injury, genetic predisposition, and mechanical loading from occupational activities or high-impact sports [3]. 

A variety of treatment strategies are available for knee osteoarthritis, including both conservative and surgical interventions [4]. Among the conservative therapies, non-steroidal anti-inflammatory drugs (NSAIDs), intra-articular injections with corticosteroids, and hyaluronic acid (HA) have been used to manage mild to moderate knee OA [5]. HA, which is a key component of synovial fluid, provides a viscoelastic response to forces in the joint, thereby facilitating joint movement and providing mechanical shock absorption [6,7]. There are several HA formulations available for clinical use, varying in molecular weight, cross-linking, source (avian-derived or biofermented), and concentration. These variations influence their rheological properties, joint residence time, and therapeutic efficacy [8]. 

In recent years, intra-articular platelet-rich plasma (PRP) has gained attention for its potential to modulate the joint environment and promote healing. The clinical application of PRP is based on the ability of platelets to release a range of essential growth factors (e.g., TGF-β1, IGF-1, FGF-2, VEGF, and PDGF) and cytokines in an orchestrated manner, thereby providing a regenerative stimulus that accelerates and promotes tissue repair [5,9,10]. In addition to the production of these anabolic growth factors, the use of leukocyte-poor PRP promotes anti-inflammatory effects and may help lower inflammation in the synovial tissue and reduce pain and swelling in the joint [11,12]. This reduction in inflammation leads to a decrease in matrix metalloproteinases, which are the key enzymes involved in the degradation of the cartilage extracellular matrix [13]. 

The combination of HA and PRP has shown promise in promoting both mechanical and biological benefits through different mechanisms [13], making the combination advantageous without altering the fundamental characteristics of either product. Saturveithan et al. [14] compared the efficacy of HA and PRP versus HA alone in grade III–IV knee OA and reported that the combination of HA and PRP had better knee function and pain relief at 6 months after the injection, but their study did not assess the long-term effects beyond this period.

Magnetic resonance imaging (MRI) is a valuable tool for assessing bone marrow lesions (BMLs), cartilage lesions, and synovitis changes prior to and after treatment [15]. Thus far, no studies have evaluated the structural changes on MRI for knee OA undergoing combination treatment with HA and PRP.

We hypothesize that the combination of HA+PRP is more effective than HA alone in improving pain, functional outcomes, and structural parameters across all time points in patients with knee osteoarthritis.

## 2. Materials and Methods

### 2.1. Study Design

**Figure 1:**
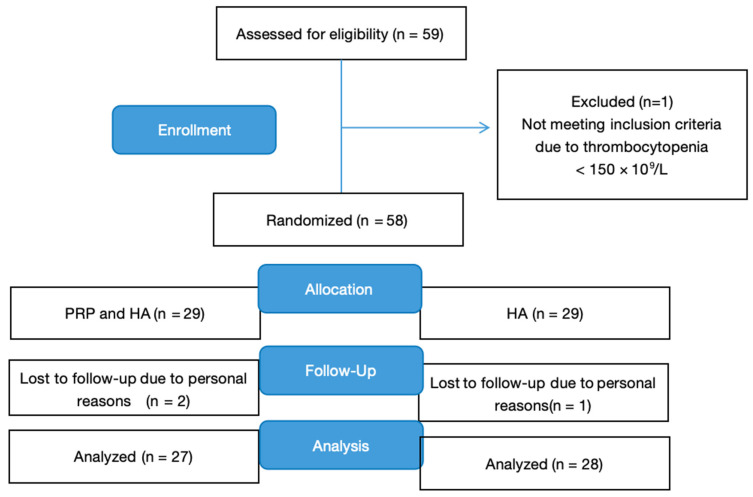
Flowchart of participant assessment, randomization, allocation, follow-up, and analysis. HA, hyaluronic acid; PRP, platelet-rich plasma.

This study was conducted as a randomized, controlled, double-blind clinical trial at an outpatient specialist medical center according to the CONSORT guidelines (Figure 1). The inclusion criteria were based on specific criteria for patients with knee osteoarthritis (OA), and a total of fifty-eight patients were prospectively recruited.

The inclusion criteria were patients aged between forty and eighty years old, suffering from femoro-tibial knee OA, Kellgren and Lawrence OA grade 2–3 based on knee radiographs less than six months old, symptomatic knee OA characterized by a walking pain VAS score of ≥40 mm on a 0 to 100 mm scale, outpatients capable of walking 50 m without assistance, and those capable of understanding the study’s imperatives and written instructions. The exclusion criteria included recent viscosupplementation or corticosteroid injection at the treatment site within the past three months, patients suffering from patellofemoral osteoarthritis only, the use of non-steroidal anti-inflammatory drugs within the past seven days, prior PRP or PRP/HA injection in the past twelve months, thrombocytopenia (<150 × 109/L), any planned knee surgery within the next twelve months, unstable injuries such as acute meniscus injury, history of allergy to HA, and rheumatological or other autoimmune disorders. This study was approved by the SingHealth Centralised Institutional Review Board, an ethics committee in Singapore (approval number: 2020/2035, 12 July 2021).

### 2.2. Randomization and Masking

In this study, eligible subjects were randomly assigned to either the experimental (HA+PRP) or control (HA) group through the use of a randomization table and envelope prepared by the research coordinator. This method of allocation concealment prevents selection bias by ensuring a balanced ratio of 1:1. To minimize the risk of bias, a double-blinded approach was employed. Participants, sports physicians performing the injections, assessors, research coordinators, and the radiologist remained blinded to the group allocation and analysis throughout the study.

### 2.3. Participants

Potential participants were screened by sports physicians and underwent a clinical evaluation and full blood count prior to recruitment. They were also sent for knee radiographs if they did not have a valid scan less than six months old. The nature of the study, procedures involved, expected duration, and potential risks and benefits were discussed with the participants. Informed consent was obtained prior to the start of the study, and participants were informed that participation was entirely voluntary. They could withdraw from the study at any time without affecting subsequent medical assistance and treatment. Eligible participants were required to complete baseline questionnaires, including the VAS pain score, WOMAC, and 5Q-5D-5L. These tools are used to assess pain, physical function, health-related quality of life, and treatment response, respectively. Follow-up questionnaires were scheduled at month 1, month 2, month 6, and month 12 after the first treatment. Participants also underwent MRI of the affected knee at baseline and again at month 12. Both groups received two intra-articular knee injections at two- to four-week intervals using a lateral patellofemoral approach with the knee in 30-degree flexion. The injections were administered by experienced sports physicians with at least five years of sports medicine practice using an aseptic technique. To maintain blinding, all participants underwent blood withdrawal regardless of their treatment group. The injectate was prepared in a separate room and then delivered to the procedure room. A screen was placed in front of the patient to prevent participants from seeing the procedure, from blood withdrawal to knee intra-articular injection. The primary outcome was the change in VAS at 12 months. Secondary outcomes included WOMAC, EQ-5D-5L, and MRI-based WORMS measures at the specified time points.

### 2.4. Intervention

#### 2.4.1. The HA+PRP Group

The experimental group received a combination of autologous HA+PRP prepared with a dedicated medical device using Cellular Matrix A-CP HA (Regen Lab SA, Le Mont-sur-Lausanne, Switzerland). Cellular Matrix A-CP HA consists of sterile vacuum blood collection tubes (A-CP HA tubes), each containing 2 mL of a non-crosslinked HA gel (1550 kDa) at 20 mg/mL from bacterial fermentation, a polymeric separator gel, and an anticoagulant solution of sodium citrate. The A-CP HA tubes allow for automated collection of 6 mL of blood and PRP preparation in a closed circuit. The separator gel is designed for platelets and plasma isolation due to its specific density and thixotropic ability. This gel becomes fluid when subjected to physical stress under 1500× *g* of centrifugal force and regains its original firm consistency when the centrifuge stops. Blood components are separated according to their specific density with the separator gel migrating upward and inserting itself precisely below the plasma and platelets. At the end of the centrifugation, the separator gel forms a physical barrier that retains erythrocytes and pro-inflammatory white blood cells in the lower part of the tube. The resulting PRP (approximately 3 mL) has a platelet concentration factor of 1.5–1.6 times higher than the baseline in blood and is poor in leukocytes. The PRP is combined with the HA gel (approximately 2 mL) gel by gentle agitation of the tube to form the PRP and HA mix, resulting in a total volume of ~5 mL.

#### 2.4.2. The HA Group

The control treatment used was ORTHOVISC (Anika Therapeutics, Inc., Bedford, MA, USA), which is a 2 mL non-crosslinked, high-molecular-weight HA gel. ORTHOVISC is composed of 15 mg/mL sodium hyaluronate obtained from bacterial fermentation and physiological phosphate buffer. The molecular weight range of ORTHOVISC is 1000–2900 kDa. ORTHOVISC was chosen as a control because it was available in the clinical inventory and its molecular weight was the closest among the stocked HA formulations to that of the HA found in the Cellular Matrix A-CP-HA kit.

### 2.5. MRI Outcomes

MRI was performed using clinical 1.5T MRI scanners. These included a GE Healthcare scanner (Chicago, IL, USA) equipped with an 8-channel T/R or a 4-channel T/R knee coil, and a Philips Healthcare scanner (Best, The Netherlands) equipped with a 16-channel T/R knee coil. The imaging protocol included sagittal, coronal, and axial fat-suppressed 2D fast spin-echo (FSE) proton-density weighted sequences, and a sagittal high-resolution intermediate-weighted 2D FSE sequence. These sequences are designed to provide detailed images of the knee joint, which can be used to assess various structures such as cartilage, ligaments, soft tissues, and bones.

A board-certified musculoskeletal radiologist with 20 years of experience assessed the MRI by applying the Whole-Organ Magnetic Resonance Imaging Score (WORMS) method [16]. WORMS provides a comprehensive assessment of the knee joint, dividing the knee into 15 different regions. The BML score is measured in the T2 image with grade 0 indicating normal conditions with no presence of BML, grade 1 affecting less than 33% of the subregion, grade 2 affecting 33–66% of the subregion, and grade 3 corresponding to more than 66% of the subregion. The total score is the sum of the regional scores.

## 3. Statistical Analysis

### Sample Size Calculation

Based on the hypothesis of a minimal clinically important difference (MCID) of 20 mm on the VAS for knee OA between the HA+PRP and HA group at day 0 and month 12, we aimed for a statistical power of 80% to detect this mean difference (SD = 25) using a two-sample *t*-test [17]. This calculation indicated that a total of 52 subjects would be required. Considering an attrition rate of 10%, we planned to recruit twenty-nine patients per arm, resulting in a total of fifty-eight subjects for the study.

All participants were included in the analysis, adhering to the intention-to-treat principle. Categorical variables (e.g., sex, race, treatment group) were summarized as frequencies and percentages, while continuous variables (e.g., age, BMI, baseline VAS, and WOMAC scores) were reported as means and standard deviations. Baseline comparisons between the HA+PRP and HA groups were performed using chi-square tests for categorical variables and independent-samples *t*-tests for continuous variables.

Longitudinal analyses were performed using linear mixed-effects models with random intercepts to account for within-subject correlations. The primary outcome was pain, assessed using the VAS. Secondary outcomes included the WOMAC total and subscale scores (pain, stiffness, and physical function), as well as health-related quality of life measured with the EQ-5D-5L [18]. Fixed effects included treatment group, time point (baseline, 1 month, 3 months, 6 months, and 12 months), and group-by-time interaction terms. An unstructured covariance structure was assumed for repeated measures. Missing data were assumed to be missing at random (MAR) and were handled using maximum likelihood estimation without imputation.

Effect estimates with 95% confidence intervals were reported. Marginal effects of treatment over time were further explored with pairwise comparisons. Likelihood ratio tests were performed to compare nested models (with vs. without interaction terms) to assess the significance of the group-by-time interactions. Model assumptions, including normality of residuals and homogeneity of variance, were assessed. No adjustments for multiple comparisons were made for secondary outcomes, and these results should be interpreted as exploratory.

All statistical analyses were conducted using Stata version 17.0 (StataCorp LLC, College Station, TX, USA). A two-tailed *p*-value of <0.05 was considered statistically significant.

## 4. Results

### 4.1. Demographic Profile and Baseline Characteristics

Table 1 summarizes the demographic profiles and baseline characteristics of the participants in the HA+PRP and HA groups. The majority of participants were ethnically Chinese, with a similar distribution of male and female participants across both groups. The mean age was 57.7 years (SD = 8.0) in the HA+PRP group and 56.7 years (SD = 8.1) in the HA group. The mean BMI was similar in both groups. VAS score, WOMAC scores, EQ-5D-5L, and MRI outcomes were assessed at the beginning of the study and were comparable in both groups.

### 4.2. Primary Outcome

#### VAS

**Figure 2:**
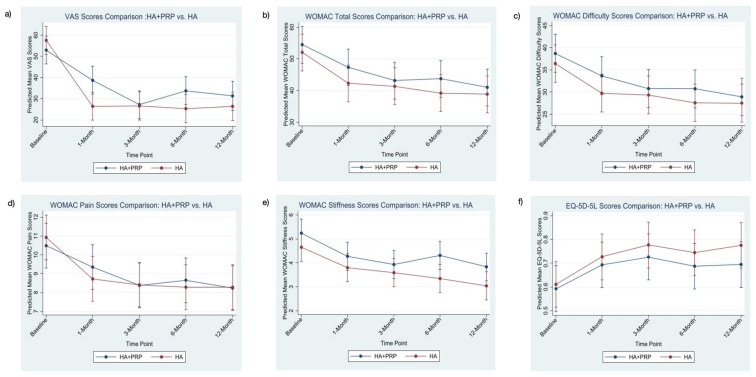
Graphical representation of Visual Analog Score (VAS), Western Ontario and McMaster Universities Osteoarthritis Index WOMAC scores and EQ-5D-5L scores of patients in the HA+PRP and HA groups over 1 year. (**a**) VAS; (**b**) WOMAC total score; (**c**) WOMAC difficulty score; (**d**) WOMAC pain score; (**e**) WOMAC stiffness score; (**f**) EQ-5D-5L score.

At 12 months, the mean change in the VAS was −21.6 (95% CI: −29.7 to −13.5) in the HA+PRP group and −31.1 (95% CI: −39.1 to −23.1) in the HA group. The between-group difference was not statistically significant (*p* = 0.102), and the observed difference did not exceed the MCID of 20 mm. The HA group exhibited a significant reduction in VAS score at 1 month (−31.1 [95% CI: −38.9 to −23.2] vs. −14.3 [95% CI: −22.2 to −6.4], *p* = 0.003) and 6 months (−32.1 [95% CI: −40.1 to −24.1] vs. −19.2 [95% CI: −27.1 to −11.3], *p* = 0.024), as compared to the HA+PRP group (Figure 2). However, the between-group differences at these timepoints did not exceed the MCID, suggesting limited clinical significance despite statistical significance.

### 4.3. Secondary Outcomes

#### 4.3.1. WOMAC and EQ-5D-5L

Both treatments resulted in a decrease in the WOMAC total scores over time, with no significant differences between the HA+PRP and HA groups at any of the assessment points (*p* > 0.05). The EQ-5D-5L scores in both groups improved over time. There were no statistically significant differences between the two groups at any of the follow-up intervals (*p* > 0.05).

#### 4.3.2. MRI Outcome Measures

**Figure 3:**
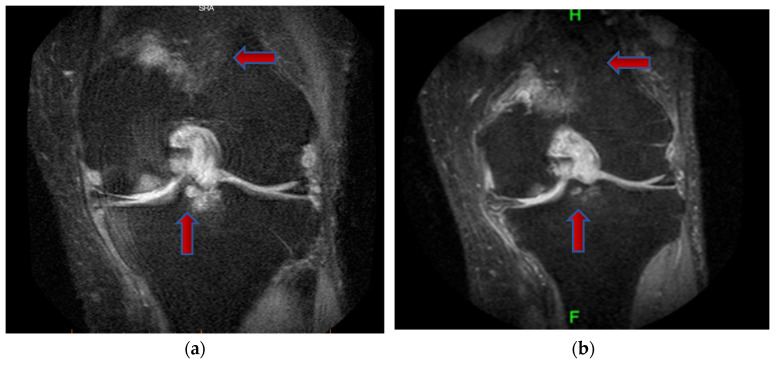
Coronal proton density-weighted fat-suppressed MRI images of the knee at (**a**) baseline and (**b**) 12 months. Red arrows indicate areas of bone marrow oedema, demonstrating a reduction in signal intensity and lesion size at follow-up.

**Figure 4:**
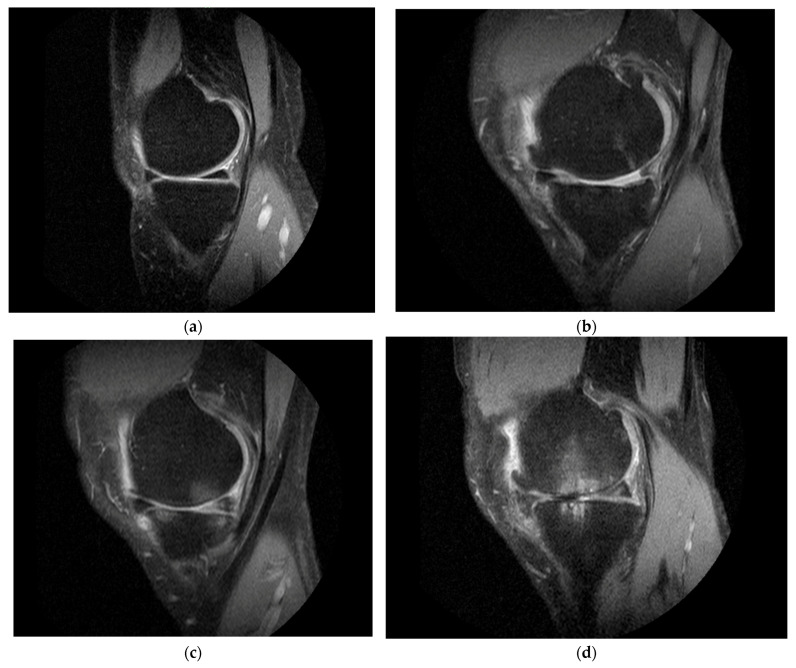
Representative T2-weighted MRI images showing bone marrow lesion grading in the knee. (**a**) Grade 0—No BML; (**b**) Grade 1; (**c**) Grade 2; (**d**) Grade 3.

This study employed MRI to assess various aspects of knee OA. The primary MRI outcome measures included total WORMS score, bone marrow edema, and synovitis of the affected knee. These measures were recorded at baseline and at 12-month follow-up (Table 2). At baseline, the total WORMS score was 77.7 for the HA+PRP group and 63.6 for the HA group. At month 12, the total WORMS score had significantly decreased to 1.2 (95% CI: −3.6 to 5.9) in the HA+PRP group and to 1.3 (95% CI: −3.5 to 6.1) in the HA group. The difference between the groups was not statistically significant (*p* = 0.962: Table 2). At baseline, both HA+PRP and HA groups had similar scores for bone marrow edema. At 12 months, MRI assessment revealed a significant decrease in bone marrow edema in the HA+PRP group (−0.7 [95% CI: −1.6 to 0.2]) compared to an increase in the HA group (0.7 [95% CI: −0.2 to 1.6], *p* = 0.030) (Figure 3 and Figure 4).

## 5. Discussion

The results of this study indicate that both HA+PRP and HA alone were effective at reducing pain and improving function in patients with grade II–III knee OA over 12 months. However, the between-group difference in VAS at 12 months—the primary outcome—was not statistically significant (*p* = 0.102) and did not exceed the MCID of 20 mm. In contrast to our initial hypothesis, the HA group demonstrated statistically significant VAS reductions at 1 and 6 months compared to HA+PRP, although these differences also did not reach the MCID threshold, limiting their clinical significance.

The current literature shows that HA+PRP is more effective than HA alone. Karasavvidis et al. [19] found that patients receiving HA combined with PRP had significantly greater improvements in pain and function compared to those receiving HA alone at 3, 6, and 12 months, and Di Martino et al. extended these findings by showing sustained clinical benefits up to 60 months [20]. Similarly, Xu et al. demonstrated that the combination of HA and PRP was superior to HA alone in inhibiting inflammation and improving pain and function over a 24-month period [21]. Marmotti et al. found that using HA as a scaffold for PRP can promote the proliferation of chondrocytes and increase the ability for cartilage repair [22]. Several possible explanations may account for the superior short-term outcomes observed in the HA group. Firstly, despite efforts to match the HA preparations based on molecular weight, the HA group received Orthovisc (1000–2900 kDa), while the HA+PRP group received a formulation of HA (1550 kDa) within the Cellular Matrix A-CP HA tubes. While both products fall within the high molecular weight category, Orthovisc contains a broader range with a higher upper limit, which may confer superior viscoelastic properties [23]. This, in turn, could enhance joint lubrication and shock absorption, contributing to greater improvements in pain relief and functional outcomes in the HA group during the early phases of the study. 

Secondly, the temporal dynamics of each treatment differ, as demonstrated in previous studies. Intra-articular HA injection for knee OA was shown to reach its peak effectiveness at six weeks [24], while PRP peak effectiveness was typically observed much later, at around three months post-injection [25]. This delayed response is due to the mechanism of action via gradual release of growth factors that mediate tissue regeneration and inflammation modulation. Additionally, the intraoperative application of leukocyte-poor PRP (LP-PRP) during arthroscopy for knee degeneration has been shown to provide short-term benefits in reducing postoperative pain and improving function and quality of life, as demonstrated in a 12-month RCT [26]. Similarly, another study evaluating the use of PRP as an adjunct to microfracture surgery for osteochondral lesions of the talus reported significant improvements in pain and function scores. The PRP group showed better pain and AOFAS scores than the microfracture-only group [27]. 

Recent studies also suggest that the platelet dosage in PRP can influence clinical outcomes in knee OA. A systematic review by Berrigan et al. found that studies with positive clinical outcomes received a mean platelet dose of 5.5 ± 474 billion platelets, compared to 2.3 ± 437 billion in studies reporting no significant benefit [28]. Similarly, Boffa et al. found that higher platelet concentrations in PRP are associated with better clinical outcomes and lower failure rates in the treatment of knee osteoarthritis [29]. Bensa et al. elucidated that while high-dosage PRP provided superior pain relief and functional improvement, low-dosage PRP still exceeded the MCID for WOMAC scores in the short to medium term [30]. These findings suggest a dose–response relationship, and it is plausible that repeated HA+PRP injections could increase the cumulative platelet dose, potentially enhancing the clinical effects [31]. In this study, meaningful improvements in pain and bone marrow lesions were achieved at 12 months from baseline following two HA+PRP injections. These results suggest the therapeutic potential of combination therapy, even at modest platelet concentrations. HA alone remains a viable option for patients with moderate knee OA, who prioritize symptom relief without additional procedural complexity.

This study utilized MRI to assess WORMS, which includes semi-quantitative radiological indicators of disease severity in knee OA. WORMS is advantageous due to its high inter-observer reliability, with intraclass correlation coefficient (ICC) values exceeding 0.80 [16], although other MRI scoring systems have also been used [15]. Raeissadat SA et al. [15], in a blinded randomized clinical trial involving grade I–III knee OA, reported significant improvement in cartilage volume in the patello-femoral compartment and synovitis in the PRP group compared to a non-PRP group. Another study evaluated the effect of PRP on articular cartilage using MRI Osteoarthritis Knee Score Bone Marrow Lesion (MOAKS BML) and found no difference in MRI parameters despite improved pain and function [32]. Similarly, Fatima et al. found no significant change in WORMS cartilage score at baseline and 1 year after PRP [33]. A prospective single-blinded study reported reduced medial tibial cartilage volume loss and a lower cartilage defect score in the HA group as compared to the control group over 2 years [34]. In this study, at 12-month follow-up, the total WORMS score between the HA+PRP and HA alone groups did not show any statistically significant differences. However, the HA+PRP group exhibited a significant reduction in bone marrow edema, while no improvement was observed in the HA alone group. The observed improvement in bone marrow edema in HA+PRP may be attributed to the anti-inflammatory and regenerative capabilities in PRP, facilitating repair by promoting the healing of micro-damage in the bone marrow and surrounding tissues [35,36,37]. To our knowledge, this is the first study to evaluate the effects of combined HA+PRP treatment on structural outcomes using MRI with WORMS.

These findings have important clinical implications. Both HA+PRP and HA treatments were effective in reducing pain and improving function in patients with knee OA over 12 months. Although HA demonstrated greater early pain relief, the addition of PRP was associated with a significant reduction in bone marrow edema at 12 months. These findings suggest potential structural benefits of HA+PRP, although clinical superiority over HA alone was not established. The use of MRI to detect structural changes reinforces the relevance of imaging biomarkers in guiding treatment decisions for knee OA. Further studies are warranted to define the clinical significance of bone marrow edema changes, optimize HA+PRP formulations, and include an extended follow-up study to assess the durability of the clinical effects and imaging changes.

### Limitations and Further Research

This study has several limitations that should be considered when interpreting the findings. Firstly, it included only patients with moderate knee OA (Kellgren and Lawrence grades 2–3), limiting generalizability to other OA severities. The single-center design and small sample size may also reduce external validity. Secondly, the outcome measures, the WOMAC score and VAS score, used in this study represent a composite total of all symptom-generating sources including both intra-articular and extra-articular factors, which may not solely relate to the effects of the intra-articular knee injection. Thirdly, a possible confounding factor affecting our clinical outcomes was the non-standardization of the physiotherapy provided to participants. Physiotherapy was arranged based on clinical discretion, which may introduce variability into the treatment outcomes. Fourthly, measurement of platelets in PRP was not performed in this study. The HA+PRP group utilized a dedicated medical device, which operates as a closed system preparation that precludes accurate quantification due to dilution effects from the HA. Given the emerging evidence of platelet dosage affecting clinical outcomes, this remains an important area for future investigations. Lastly, while a 12-month follow-up duration was sufficient to observe moderate-term interim effects, a longer follow-up is needed to fully evaluate the long-term structural and symptomatic benefits.

## Figures and Tables

**Table 1:** Participant demographics.

Characteristic	Treatment	
	HA+PRP	HA
	*n* = 29	*n* = 29
Sex, *n* (%)		
Female	13 (44.8%)	13 (44.8%)
Male	16 (55.2%)	16 (55.2%)
Ethnicity *n* (%)		
Chinese	25 (86.2%)	23 (79.3%)
Malay	1 (3.5%)	1 (3.5%)
Indian	1 (3.5%)	4 (13.8%)
Others	2 (6.9%)	1 (3.5%)
Age		
Mean (SD)	57.7 (8.0)	56.7 (8.1)
Min–max	42–77	43–73
BMI, mean (SD)	27.0 (6.1)	26.9 (3.1)
Baseline measures		
VAS, mean (SD)	52.9 (12.5)	57.5 (13.8)
WOMAC, mean (SD)		
Total	54.4 (17.1)	52.0 (19.0)
Pain	10.5 (3.3)	10.9 (3.9)
Stiffness	5.2 (1.6)	4.7 (1.9)
Difficulty	38.7 (13.1)	36.4 (14.1)
EQ-5D-5L, mean (SD)	3.9 (3.2)	3.3 (2.8)
WORMS score (SD)		
Total score	77.7 (44.8)	63.6 (43.2)
Bone marrow edema	5.5 (4.7)	5.7 (4.4)
Synovitis	1.6 (0.7)	1.6 (0.7)

Abbreviations: HA, hyaluronic acid; PRP, platelet-rich plasma; BMI, body mass index; VAS, visual analog score; WOMAC, Western Ontario and McMaster Universities Osteoarthritis Index; EQ-5D-5L: EuroQol 5-Dimension 5-Level; WORMS, Whole-Organ MRI Score; SD, Standard Deviation.

**Table 2:** Linear mixed-effects models for WOMAC, EQ-5D-5L, and WORMS scores.

Predictor	Treatment		
	HA+PRP (*n* = 29)	HA (*n* = 29)	*p*-Value
**WOMAC total score (95% CI)**			
At baseline	54.4	52	
Month 1	(−)7.1 (−13.4 to −0.86)	(−)9.8 (−16.0 to −3.5)	0.563
Month 2	(−)11.3 (−17.6 to −5.1)	(−)10.7 (−16.9 to −4.4)	0.885
Month 6	(−)10.8 (−17.0 to −4.5)	(−)12.9 (−19.1 to −6.6)	0.642
Month 12	(−)13.4 (−19.7 to −7.2)	(−)13.2 (−19.5 to −6.9)	0.957
**WOMAC pain score (95% CI)**			
At baseline	10.5	10.9	
Month 1	(−)1.1 (−2.5 to 0.2)	(−)2.2 (−3.5 to −0.8)	0.267
Month 2	(−)2.1 (−3.4 to −0.8)	(−)2.5 (−3.9 to −1.2)	0.667
Month 6	(−)1.8 (−3.2 to −0.5)	(−)2.7 (−3.9 to −1.3)	0.390
Month 12	(−)2.2 (−3.6 to −0.9)	(−)2.7 (−3.9 to −1.3)	0.667
**WOMAC stiffness score (95% CI)**			
At baseline	5.2	4.7	
Month 1	(−)0.9 (−1.6 to −0.3)	(−)0.9 (−1.5 to −0.2)	0.826
Month 2	(−)1.3 (−1.9 to −0.7)	(−)1.1 (−1.7 to −0.4)	0.608
Month 6	(−)0.9 (−1.6 to −0.3)	(−)1.3 (−1.9 to −0.7)	0.420
Month 12	(−)1.4 (−2.1 to −0.8)	(−)1.6 (−2.3 to −0.9)	0.660
**WOMAC difficulty score (95% CI)**			
At baseline	38.7	36.4	
Month 1	(−)5.0 (−9.6 to −0.4)	(−)6.7 (−11.3 to −2.1)	0.618
Month 2	(−)7.9 (−12.5 to −3.3)	(−)7.1 (−11.7 to −2.5)	0.803
Month 6	(−)8.0 (−12.6 to −3.4)	(−)8.9 (−13.5 to −4.3)	0.787
Month 12	(−)9.8 (−14.4 to −5.2)	(−)8.9 (−13.5 to −4.3)	0.795
**EQ-5D-5L score (95% CI)**			
At baseline	3.9	3.3	
Month 1	0.1 (−0.002 to 0.2)	0.1 (0.01 to 0.2)	0.835
Month 2	0.1 (0.03 to 0.2)	0.2 (0.1 to 0.3)	0.667
Month 6	0.1 (−0.01 to 0.2)	0.1 (0.03 to 0.2)	0.600
Month 12	0.1 (−0.001 to 0.2)	0.2 (0.1 to 0.3)	0.395
**Total WORMS score (95% CI)**			
At baseline	77.7	63.6	
Month 12	1.2 (−3.6 to 5.9)	1.3 (−3.5 to 6.1)	0.962
**Bone marrow edema (95% CI)**			
At baseline	5.5	5.7	
Month 12	(−)0.7 (−1.6 to 0.2)	0.7 (−0.2 to 1.6)	**0.030**
**Synovitis**			
At baseline	1.6	1.6	
Month 12	(−)0.04 (−0.1 to 0.1)	(−)0.03 (−0.1 to 0.1)	0.973

Abbreviations: HA, hyaluronic acid; PRP, platelet-rich plasma; WOMAC, Western Ontario and McMaster Universities Osteoarthritis Index; CI, confidence interval.

## Data Availability

[Clinical Efficacy of Platelet-Rich Plasma and Hyaluronic Acid Versus Hyaluronic Acid for Knee Osteoarthritis with MRI Analysis] [https://clinicaltrials.gov/study/NCT06636695?term=NCT06636695&rank=1] [NCT06636695] (accessed on 18 May 2025).
